# *ABCC2* Genetic Variation and Vincristine-Induced Peripheral Neuropathy Risk in Mexican Pediatric Patients with Acute Lymphoblastic Leukemia

**DOI:** 10.3390/ijms27219998

**Published:** 2026-11-08

**Authors:** María Fernanda Monroy-Baroja, Carlos Galaviz-Hernandez, Laura María García-Torres, Elio Aarón Reyes-Espinoza, Luis Felipe Cuellar-Guzman, Lourdes Patricia Córdova-Hurtado, Laura Jazel Barragan-Zuñiga, Martha Sosa-Macías

**Affiliations:** 1 Centro Interdisciplinario de Investigación para el Desarrollo Integral Regional Unidad Durango (CIIDIR Durango), Instituto Politécnico Nacional, Durango 34220, Durango, Mexico; mariafermonroy_@hotmail.com (M.F.M.-B.); cgalaviz@ipn.mx (C.G.-H.); 2 Centro Estatal de Cancerología de Durango (CECAN), Secretaria de Salud del Estado de Durango, Durango 34000, Durango, Mexico

**Keywords:** acute lymphoblastic leukemia, vincristine, peripheral neuropathy, pharmacogenetics, *ABCC2*, pediatric oncology, Mexican population

## Abstract

Vincristine-induced peripheral neuropathy (VIPN) is a common dose-limiting toxicity in pediatric acute lymphoblastic leukemia (ALL), and pharmacogenetic evidence in admixed populations remains limited. We evaluated variants in *ABCC1*, *ABCC2*, *ABCB1*, *CYP3A4*, *CYP3A5*, and *CEP72* in Mexican pediatric patients with ALL using an ambispective case–control design (n = 43; aged 2–18 years). VIPN was graded using CTCAE v4.0; cases were defined as grade ≥ 2 (n = 11) and controls grade 0–1 (n = 32). Nine single nucleotide variants (SNVs) were genotyped by allele-specific qPCR and analyzed under codominant, dominant, recessive, and log-additive models, including haplotype analyses. VIPN occurred in 25.6% of patients, with 90.9% of cases occurring during induction (*p* = 0.001). *ABCC2* rs11190298 was nominally associated with VIPN under codominant (OR = 0.21; 95% CI: 0.04–0.97), dominant (OR = 0.15; 95% CI: 0.03–0.71), and log-additive (OR = 0.17; 95% CI: 0.04–0.66) models, and *ABCC2* haplotypes showed a nominal global association (*p* = 0.003). These associations did not withstand correction for multiple testing. No significant associations were found for *ABCC1*, *ABCB1*, *CYP3A4*, *CYP3A5*, or *CEP72*. Early-onset toxicity may reflect differences in drug exposure or treatment intensity, which pharmacokinetic data could clarify. These hypothesis-generating findings require replication in larger prospective cohorts.

## 1. Introduction

Acute lymphoblastic leukemia (ALL) is the most common pediatric malignancy worldwide, accounting for approximately 75–80% of all childhood leukemias [1]. In Mexico, leukemias represent more than half of pediatric cancers, with ALL as the predominant subtype and an estimated incidence of 73 cases per million children annually [2].

Vincristine (VCR) remains a cornerstone of ALL treatment due to its potent antimitotic activity, mediated by binding to tubulin heterodimers and disrupting microtubule polymerization [3]. Despite its therapeutic efficacy, VCR is frequently associated with vincristine-induced peripheral neuropathy (VIPN), a dose-limiting toxicity characterized by sensory and motor dysfunction, including paresthesia, neuropathic pain in the extremities, and muscle weakness, which can substantially impair quality of life and compromise treatment adherence [4].

Interindividual variability in VIPN susceptibility is only partially explained by clinical factors, and genetic determinants have emerged as important contributors. From a pharmacokinetic perspective, VCR is primarily metabolized by the hepatic enzymes CYP3A5 and CYP3A4 [5]. The *CYP3A5* rs776746 variant affects enzyme expression and has been associated with interindividual differences in drug exposure [6,7]. Despite its limited metabolic contribution, patients carrying functional *CYP3A4* alleles have shown a lower risk of developing VIPN than those with non-functional variants [8], and the protective effect observed among carriers of the CYP3A4*1B variant (rs2740574) and CYP3A5*3 has been proposed to reflect differences in VCR exposure [9].

Drug disposition is further modulated by ATP-binding cassette (ABC) transporters, which mediate vincristine efflux across hepatocytes and the blood–nerve barrier. Variants in *ABCC1* (rs3743527) and *ABCC2* (rs11190298, rs3740066) have been associated with altered transporter activity and VIPN risk in pediatric cohorts [10], whereas the *ABCB1* variants rs1045642 and rs2032582, encoding P-glycoprotein, could theoretically alter systemic and neuronal drug exposure through changes in transporter expression and efflux efficiency [11,12].

Beyond drug disposition, pharmacodynamic variability may also contribute to VIPN susceptibility. The *CEP72* gene encodes a centrosomal protein involved in microtubule organization. The promoter variant rs924607 (TT genotype) has been associated with increased VIPN risk during maintenance therapy [13], while the missense variant rs12522955 has been implicated in VIPN in patients with brain tumors [14].

Despite these advances, pharmacogenetic findings remain inconsistent, and data from admixed populations are scarce. The Mexican population exhibits substantial genetic admixture, which may influence allele frequencies, linkage disequilibrium patterns, and gene–drug interactions [15,16]. To date, pharmacogenetic determinants of VIPN have not been comprehensively evaluated in Mexican pediatric patients with ALL.

This study aimed to assess the association between selected single nucleotide variants (SNVs) in *ABCC1*, *ABCC2*, *ABCB1, CYP3A4*, *CYP3A5* and *CEP72* genes and the development of VIPN in this population, contributing to population-specific data to inform risk stratification and personalized therapy. In this exploratory analysis, *ABCC2* rs11190298 showed a nominal association with VIPN, whereas variants in the remaining genes showed no association.

## 2. Results

### 2.1. Patient Characteristics

A total of 43 patients were included, with a median age of 5.5 years; 60.5% were male. Eleven patients (25.6%) developed VIPN and were classified as cases, while the remaining 32 (74.4%) were classified as controls. No statistically significant differences were observed between groups in age, sex, anthropometric measurements, nutritional status, or cumulative VCR dose during induction (Table 1). Likewise, no significant differences were detected in baseline liver function parameters or in treatment-associated relative changes (%Δ) in these markers (Figure S1).

A significant difference was observed in treatment phase distribution between groups (*p* = 0.001), with 90.9% of cases occurring during the induction phase, compared with a lower proportion among controls. Dexamethasone, intrathecal chemotherapy, and ondansetron were the most frequently co-administered drugs in both cases (8/11, 6/11, 6/11) and controls (26/32, 19/32, 18/32), with no statistically significant differences between groups (Fisher’s exact test: dexamethasone *p* = 0.672, intrathecal chemotherapy *p* = 1.00, ondansetron *p* = 1.00).

### 2.2. VIPN Characteristics

Among affected patients, neuropathy severity was distributed as follows: grade 2 (27.3%), grade 3 (54.5%), and grade 4 (18.2%). Neuropathic pain in the lower extremities was the most prevalent symptom (100%) and frequently required pharmacological management. Constipation was observed in 90.9% of cases, suggesting involvement of autonomic nerve fibers. Additional manifestations included lower limb weakness and jaw pain (27.3% each), consistent with the known neurotoxic profile of VCR.

### 2.3. Genotype Analyses

No significant differences in allele or genotype frequencies were observed between cases and controls for the evaluated variants in *ABCC1*, *ABCB1*, *CYP3A4*, *CYP3A5* or *CEP72* under any inheritance model. In contrast, the *ABCC2* rs11190298 variant showed a nominally significant difference in allele frequency (*p* = 0.015), with the G allele more frequent among controls than cases (53.1% vs. 22.7%). The AA genotype was significantly more prevalent in cases than in controls (54.5% vs. 15.6%; *p* = 0.018) (Table 2).

For *ABCC2* rs3740066, no significant genotype-level differences were observed; however, a trend toward association was noted in allele frequency (*p* = 0.078), with the T allele more frequent in controls (45.3% vs. 22.7%). No variant showed significant deviation from Hardy–Weinberg equilibrium in the control group (exact test; Table 2), although this test has limited power with low genotype counts.

Association analyses showed that carriers of the rs11190298 G allele had nominally lower odds of developing VIPN under codominant (OR = 0.21; 95% CI: 0.04–0.97; *p* = 0.013), dominant (OR = 0.15; 95% CI: 0.03–0.71; *p* = 0.014), and log-additive (OR = 0.17; 95% CI: 0.04–0.66; *p* = 0.004) inheritance models (Table 3). Similarly, rs3740066 was nominally associated with VIPN risk under the log-additive model only (OR = 0.28; 95% CI: 0.08–0.99; *p* = 0.034). No significant associations were identified for *ABCC1*, *ABCB1*, *CYP3A5* or *CEP72*, or under any inheritance model (Table S1). For *CYP3A4* rs2740574, analysis was not feasible because no patients carried the CC genotype among either cases or controls. These associations are nominal. Across the 82 comparisons performed, approximately four nominally significant results would be expected by chance at *p* < 0.05, and none reached the Bonferroni-adjusted threshold (*p* < 0.05/82 ≈ 0.0006). The *ABCC2* findings should therefore be considered exploratory and hypothesis-generating.

### 2.4. Haplotype Analyses

Analysis of *ABCC2* revealed moderate linkage disequilibrium between rs11190298 and rs3740066 (GT haplotype: D’ = 0.60, r = 0.54, *p* < 0.001) and a nominal global association with VIPN (*p* = 0.003). The G-T and G-C haplotypes were also associated with decreased VIPN risk relative to the reference haplotype (A-C) (Table 4). The odds ratios close to zero with wide confidence intervals for the individual *ABCC2* haplotypes most likely reflect sparse data, as very few cases carried these haplotypes. Because the two variants are in linkage disequilibrium, these estimates are not independent and should not be interpreted as independent or additive protective effects. In contrast, *ABCB1* variants (rs2032582 and rs1045642) formed a robust linkage disequilibrium block (D’ = 0.94, r = −0.84, *p* < 0.001), but the resulting haplotype distribution was not associated with VIPN (global *p* = 0.25; Table S2). For *CYP3A* variants (rs776746 and rs2740574), no evidence of linkage disequilibrium was observed (D’= 0.35, r = 0.16, *p* = 0.128), and global haplotype analysis showed no association with VIPN (global *p* = 0.50; Table S3).

Finally, no significant haplotype association was observed for *CEP72* (rs924607 and rs12522955), despite strong linkage disequilibrium between these variants (D’ = 0.99, r = −0.29, *p* = 0.007; Table S4).

## 3. Discussion

This exploratory study identified a nominal association between *ABCC2* rs11190298 and susceptibility to VIPN in a cohort of Mexican pediatric patients with ALL, with the G allele associated with lower odds of VIPN across several inheritance models. To our knowledge, this is the first pharmacogenetic study of VIPN conducted in a pediatric population from northern Mexico. The G allele frequency observed in controls (53.1%) is consistent with frequencies reported in Latin American reference populations (46–52%), as catalogued in gnomAD and Ensembl, which supports the quality of genotyping and the representativeness of the control group, although it does not exclude population stratification, since genetic ancestry was not assessed.

The observed VIPN frequency of 25.6% is consistent with rates reported in pediatric cohorts in Spain and the United States (26.8–32.9%), though lower than estimates from a Finnish cohort (96%) [10,13,17]. Within Mexico, comparable frequencies have been reported using neurophysiological assessments [18], whereas higher estimates have been described with clinical scoring instruments such as the ped-mTNS [19], likely reflecting differences in diagnostic sensitivity rather than true biological variation. No significant associations were observed between VIPN and clinical variables, including age, sex, nutritional status, cumulative VCR dose during induction, or liver function parameters, consistent with prior evidence that clinical factors alone are insufficient to explain interindividual susceptibility [12,20,21,22].

Most VIPN cases (90.9%) occurred during the induction phase. Early-onset toxicity has been associated with variability in drug exposure, whereas late-onset toxicity may reflect cumulative pharmacodynamic effects on neuronal integrity [23]. However, the timing of neuropathy alone is insufficient to establish a pharmacokinetic mechanism, as no vincristine concentrations were measured. Alternative explanations include higher treatment intensity during induction, concomitant medications (e.g., corticosteroids), closer clinical monitoring, and differences in the timing and frequency of neuropathy assessments. Treatment phase differed significantly between cases and controls and may therefore act as a confounder.

The association between *ABCC2* rs11190298 and VIPN is biologically plausible. *ABCC2* encodes MRP2, a key mediator of hepatobiliary VCR excretion [24]. We hypothesize that the G allele may enhance MRP2 expression or transport function, promoting biliary excretion of VCR and thereby limiting systemic and intracellular drug exposure, consistent with the protective effect observed among G-allele carriers in this cohort. This mechanism is speculative: no functional or pharmacokinetic data were obtained in this study, and it requires independent replication and experimental validation. Conversely, the A allele may be associated with reduced transporter activity, resulting in greater VCR exposure and enhanced neurotoxicity, consistent with the higher frequency of the AA genotype observed among cases and with prior findings in pediatric ALL cohorts [10]. Although the rs11190298 variant is located in a downstream regulatory region and its functional consequences have not yet been established in vitro or in animal models, it may influence gene expression either directly or through linkage disequilibrium with functional variants that affect transporter activity. The log-additive association observed for rs3740066, together with the significant global haplotypes association, are compatible with a possible role of combined *ABCC2* variation in VIPN susceptibility. However, the haplotype estimates are imprecise and should be interpreted with caution. It should be noted that previous pharmacogenetic studies have reported null associations for candidate variants in transporter genes and VIPN [11,12], and the novelty of our finding should be weighed against the small sample size and the absence of replication.

Among the remaining pharmacokinetic candidates evaluated, none showed a significant association with VIPN. These null results should not be interpreted as evidence of no association: with 43 patients and no formal power calculation, the study was underpowered to detect modest effects. In addition, only selected, previously reported candidate variants were genotyped, and genetic variation in the corresponding genes was not comprehensively evaluated. The absence of an *ABCC1* rs3743527 association is consistent with prior reports [10]. For *CYP3A5* rs776746, the high prevalence of the *3/*3 non-expressor genotype in this cohort likely constrained statistical power to detect an effect, despite this gene’s established role in VCR metabolism [5]. Similarly, no association was observed for CYP3A4*1B rs2740574 or for the *ABCB1* variants rs2032582 and rs1045642. Although an early report suggested a potential protective effect of the CYP3A4*1B variant against peripheral neuropathy, subsequent candidate-gene and pharmacokinetic evaluations in pediatric cohorts failed to confirm any significant impact of this variant on vincristine clearance, exposure, or neurotoxicity [9,25]. Likewise, despite the established role of P-glycoprotein as a primary efflux transporter for vincristine, functional variants in *ABCB1*, specifically rs1045642 and rs2032582, have consistently shown no significant correlation with vincristine pharmacokinetic parameters, motor performance deficits, or VIPN development across diverse pediatric populations [11,12,26,27]. Among the variants examined, *ABCC2* rs11190298 showed the only nominal association, which requires confirmation in independent cohorts.

In contrast to the pharmacokinetic genes discussed above, *CEP72* acts through a pharmacodynamic mechanism, encoding a centrosomal protein involved in microtubule organization. No significant association was observed for either *CEP72* variant (rs924607 or rs12522955) in this cohort. Given that the predominance of VIPN in our sample occurred during induction, this may have limited the detectability of pharmacodynamic variants whose influence appears more relevant during maintenance therapy, when cumulative neurotoxicity predominates [28].

The Mexican population is characterized by substantial genetic admixture, with variable proportions of Indigenous American, European, and African ancestries, which influences allele frequencies and linkage disequilibrium patterns in ways that may affect the replicability of pharmacogenetic findings across populations [29]. Although control-group allele frequencies were similar to those in Latin American reference databases, this does not exclude population stratification. Genetic ancestry was not assessed or estimated in our cohort; however, it was verified that all the children’s parents and grandparents belonged to the Mexican-mestizo population. All participants were Mexican children from the state of Durango treated at CECAN, so generalization to other Mexican and Latin American populations should be made with caution. From a translational perspective, *ABCC2* rs11190298 represents a biologically plausible candidate for VIPN risk stratification; however, clinical implementation is premature pending replication in adequately powered cohorts.

This study has several limitations. The small sample size restricts statistical power and the precision of effect estimates, and no formal power analysis was performed. No correction for multiple testing was applied given the exploratory nature of the study; the *ABCC2* association is therefore nominal and hypothesis-generating. The ambispective design introduces potential outcome misclassification, particularly between grade 0–1 and grade ≥ 2 neuropathy. Because retrospective records may be incomplete, under-recording of mild or moderate neuropathy, and thus misclassification of some cases as controls, cannot be excluded. As the classifying clinician was blinded to genotype, such misclassification would be expected to be unrelated to genotype and to bias associations toward the null. The absence of pharmacokinetic and functional data limits mechanistic interpretation. Cumulative VCR exposure was limited to the prescribed induction doses and was not calculated up to the date of neuropathy onset. The lack of multivariable adjustment precludes control for potential confounders, including treatment phase and treatment protocol, and the distribution of protocols between cases and controls was not formally compared. Genetic ancestry was not assessed, so population stratification cannot be excluded. Finally, only previously reported candidate variants were analyzed; unknown or untested variants related to VIPN may therefore have been missed.

## 4. Materials and Methods

### 4.1. Study Design and Population

An ambispective case–control study was conducted in 43 Mexican pediatric patients (aged 2–18 years) diagnosed with ALL between October 2023 and December 2025 at the Durango State Cancer Center (CECAN), Mexico. All participants were children from the state of Durango; genetic ancestry was not determined. Clinical data on VIPN were retrospectively reconstructed from medical records in 34 patients and prospectively collected for 9, while biological sampling for genotyping was performed prospectively in all participants.

Patients were treated according to the St. Jude Total Therapy protocols XIII B (n = 9), XV (n = 28), and XVII (n = 6). In all protocols, VCR was administered intravenously at 1.5 mg/m^2^ (maximum 2 mg per dose) weekly for four doses during induction, followed by 6 to 16 additional doses administered weekly or monthly in later phases, depending on risk group and treatment phase. Patients receiving azole antifungals were excluded because of their inhibitory effect on CYP enzymes.

The study was approved by the Research and Ethics Committees of the General Hospital of Durango, Mexico (registration number 611/023), and was conducted in accordance with the Declaration of Helsinki and the Mexican General Health Law. Written informed consent was obtained from legal guardians and assent was obtained from patients aged ≥ 7 years.

### 4.2. Clinical and Demographic Data Collection

Clinical and demographic variables extracted from medical records included age, sex, weight, height, cumulative VCR dose during induction (mg; sum of the four prescribed doses), and treatment phase (induction vs. post-induction). Nutritional status was classified according to Centers for Disease Control and Prevention (CDC) growth charts into underweight, normal weight, overweight, or obese categories.

Liver function parameters, including aspartate transaminase (AST), alanine transaminase (ALT), alkaline phosphatase (AP), total, direct, and indirect bilirubin, total proteins and albumin, were recorded at two time points: the measurement closest to the start of the induction phase (baseline) and the measurement closest to the end of the induction phase (post-induction). To standardize the analysis and minimize the influence of interindividual baseline variability, absolute (Δ) and relative percentage (%Δ) changes from baseline were calculated for each parameter.

Concomitant medications administered on the same day as VCR were recorded to evaluate potential drug–drug interactions.

### 4.3. VIPN Assessment

Peripheral neuropathy was evaluated using a structured clinical questionnaire (File S1) combined with a standardized neurological examination. Assessed symptoms included numbness or tingling in the extremities, neuropathic pain, constipation, jaw pain, hoarseness, and decreased peripheral muscle strength. Severity was graded according to the Common Terminology Criteria for Adverse Events (CTCAE), version 4.0. Consistent with the established pharmacogenetic literature [13,20,30], patients were classified as cases if they developed grade ≥ 2 neuropathy and as controls if they presented grade 0–1; the highest grade documented during VCR treatment was used.

The same CTCAE criteria were applied to all patients, regardless of the data collection method. For the 34 retrospectively evaluated patients, all information related to neuropathy (symptoms, examination findings, and analgesic requirements) was transcribed verbatim from the clinical records, together with the date of registration. Patients in whom no neuropathy was documented in the clinical record were classified as controls; no data imputation was performed. For the 9 prospectively evaluated patients, the structured questionnaire and neurological examination were applied directly. To minimize inter-observer variability, all clinical assessments and case–control classifications were performed or reviewed by a single clinician, who classified patients using only clinical information and was blinded to genotyping results.

### 4.4. DNA Extraction and Genotyping

Genomic DNA was extracted from peripheral blood samples collected in EDTA tubes using a previously described method [31]. DNA integrity was confirmed by agarose gel electrophoresis, and concentration and purity were assessed using a Nanodrop™ spectrophotometer (Thermo Fisher Scientific, Waltham, MA, USA). Samples were stored at −20 °C until analysis.

Nine SNVs were genotyped by allele-specific quantitative PCR on a StepOne™ Real-Time PCR System (Applied Biosystems™,Foster City, CA, USA). *ABCC1* rs3743527 and *CYP3A5* rs776746 were genotyped using the TaqMan^®^ SNP Genotyping Assay (C__8934057_30 and C_26201809_30, respectively; Applied Biosystems™). *ABCC2, ABCB1*, *CYP3A4* and *CEP72* variants, including *ABCC2* rs11190298 (Hs.GT.rs11190298.G.1), *ABCC2* rs3740066 (Hs.GT.rs3740066.G.1), *ABCB1* rs2032582 (CD.GT.YRYC9183.1), *ABCB1* rs1045642 (Hs.ADME.rs1045642.G.1), *CYP3A4* rs2740574 (CD.GT.PFTF7270.1), *CEP72* rs924607 (Hs.GT.rs924607.T.1), and *CEP72* rs12522955 (Hs.GT.rs12522955.A.1), were genotyped using rhAmp™ SNP assays (Integrated DNA Technologies, IDT™, Coralville, IA, USA).

### 4.5. Statistical Analysis

Continuous variables are reported as median and interquartile range (IQR) and categorical variables as frequencies and percentages. Normality was assessed using the Shapiro–Wilk test. Group comparisons were performed using the Mann–Whitney U test for continuous variables and Fisher’s exact test (Pearson’s χ^2^ test when expected counts allowed) for categorical variables, as appropriate. For liver function parameters, absolute changes (Δ) and relative percentage changes (%Δ) from baseline were calculated.

Allele and genotype frequencies were determined for each SNV and compared between cases and controls using Fisher’s exact test: the allele frequencies were compared between groups, and each genotype (homozygous wild-type, heterozygous, and homozygous variant) was compared against the combination of the other two genotypes. Deviation from Hardy–Weinberg equilibrium was assessed in the control group using the exact test implemented in SNPStats [32]. Associations with VIPN were further evaluated under codominant, dominant, recessive, and log-additive inheritance models using logistic regression in SNPStats prior to its recent discontinuation (https://www.snpstats.net/start.htm (accessed on 10 August 2026)), and odds ratios (ORs), 95% confidence intervals (CIs), *p*-values, and Akaike Information Criterion (AIC) values were obtained for each model. Because of the small number of cases (n = 11), models were not adjusted for covariates.

Haplotype analyses were conducted for the variant pairs within *ABCC2* (rs11190298, rs3740066), *ABCB1* (rs2032582, rs1045642), *CYP3A* (rs776746, rs2740574), and *CEP72* (rs924607, rs12522955). Pairwise linkage disequilibrium and haplotype frequencies were estimated in SNPStats using the expectation–maximization algorithm. Associations with VIPN were evaluated by logistic regression using the most frequent haplotype as reference, with rare haplotypes (frequency < 0.01) pooled. The global haplotype *p*-value corresponds to a likelihood-ratio test comparing the model including haplotypes with the null model.

A total of 82 statistical comparisons were performed across variants, inheritance models, and haplotypes. Given the exploratory nature of the study and the limited sample size (n = 43), *p*-values are reported without correction for multiple testing, in order to limit type II error, and nominally significant results (*p* < 0.05) are distinguished from confirmatory evidence. For reference, the Bonferroni-adjusted threshold would be *p* < 0.05/82 ≈ 0.0006. No formal power calculation was performed.

Statistical significance was defined as a two-sided *p* < 0.05. Analyses other than those performed in SNPStats were conducted in Python (version 3.11.5).

## 5. Conclusions

In this exploratory study, *ABCC2* rs11190298 showed a nominal association with VIPN in Mexican pediatric patients with ALL, with the G allele associated with lower odds of neuropathy. This association did not withstand correction for multiple testing, and no association was found for the other variants evaluated in *ABCC1*, *ABCB1*, *CYP3A4*, *CYP3A5*, and *CEP72*. These findings are hypothesis-generating and require independent replication in larger, prospective cohorts integrating pharmacokinetic and functional data before any implication for individualized VCR therapy can be considered.

## Figures and Tables

**Table 1:** Clinical and demographic characteristics of pediatric patients.

Patient Characteristics	Cases (n = 11)	Controls (n = 32)	Overall (n = 43)	*p*-Value
Age (years) ^a^	4.50 (4.00, 6.65)	6.25 (4.38, 8.70)	5.50 (4.30, 8.65)	0.226 ^1^
Weight (kg) ^a^	18.50 (15.85, 32.60)	20.00 (15.00, 33.90)	20.00 (15.10, 34.80)	0.989 ^1^
Height (m) ^a^	1.08 (1.00, 1.34)	1.14 (1.02, 1.33)	1.13 (1.00, 1.34)	0.770 ^1^
Sex ^b^				
Female	5 (45.5)	12 (37.5)	17 (39.5)	0.728 ^2^
Male	6 (54.5)	20 (62.5)	26 (60.5)	
Nutritional status ^b^				
Underweight	2 (18.2)	9 (28.1)	11 (25.6)	0.516 ^2^
Normal	6 (54.5)	20 (62.5)	26 (60.5)	
Overweight	2 (18.2)	2 (6.3)	4 (9.3)	
Obese	1 (9.10)	1 (3.1)	2 (4.6)	
Cumulative VCR dose during induction (mg) ^a^	4.00 (3.60, 5.60)	4.20 (3.55, 5.30)	4.00 (3.60, 5.60)	0.576 ^1^
Stage ^b^				
Induction	10 (90.9)	10 (31.2)	20 (46.5)	0.001 ^2^
Post-induction	1 (9.1)	22 (68.8)	23 (53.5)	

^a^ Values are presented as median (interquartile range). ^b^ Values are presented as n (%). ^1^ Mann–Whitney U test; ^2^ Fisher’s exact test.

**Table 2:** Allelic and genotypic frequencies of SNVs in *ABCC1, ABCC2, ABCB1, CYP3A4, CYP3A5* and *CEP72* between cases and controls.

SNV	Allele/ Genotype	Cases n = 11	Controls n = 32	*p*-Value ^1^	HWE ^2^
*ABCC1* rs3743527 C > T	C	16 (72.7)	44 (68.7)	0.935	1.00
	T	6 (27.3)	20 (31.3)		
	CC	6 (54.5)	15 (47.0)	0.736	
	CT	4 (36.4)	14 (43.8)	0.736	
	TT	1 (9.1)	3 (9.2)	1.000	
*ABCC2* rs11190298 A > G	A	17 (77.3)	30 (46.9)	**0.015**	0.28
	G	5 (22.7)	34 (53.1)		
	AA	6 (54.5)	5 (15.6)	**0.018**	
	AG	5 (45.5)	20 (62.5)	0.480	
	GG	0 (0.0)	7 (21.9)	0.163	
*ABCC2* rs3740066 C > T	C	17 (77.3)	35 (54.7)	0.078	0.47
	T	5 (22.7)	29 (45.3)		
	CC	6 (54.5)	8 (25.0)	0.133	
	CT	5 (45.5)	19 (59.4)	0.495	
	TT	0 (0.0)	5 (15.6)	0.306	
*ABCB1* rs2032582 A > C	A	10 (45.0)	36 (56.0)	0.460	0.72
	C	12 (55.0)	28 (44.0)		
	AA	2 (18.0)	9 (28.0)	0.698	
	AC	6 (55.0)	18 (56.0)	1.00	
	CC	3 (27.0)	5 (16.0)	0.401	
*ABCB1* rs1045642 A > G	A	10 (45.0)	30 (47.0)	1.00	0.07
	G	12 (55.0)	34 (53.0)		
	AA	2 (18.0)	4 (12.0)	0.637	
	AG	6 (55.0)	22 (69.0)	0.473	
	GG	3 (27.0)	6 (19.0)	0.672	
*CYP3A4* rs2740574 C > T	C	2 (9.0)	4 (6.0)	0.643	1.00
	T	20 (91.0)	60 (94.0)		
	CC	0 (0.0)	0 (0.0)		
	CT	2 (18.0)	4 (12.0)	0.637	
	TT	9 (82.0)	28 (88.0)	0.637	
*CYP3A5* rs776746 A > G	A	6 (27.3)	16 (25.0)	1.000	1.00
	G	16 (72.7)	48 (75.0)		
	AA	1 (9.1)	2 (6.2)	1.000	
	AG	4 (36.4)	12 (37.5)	1.000	
	GG	6 (54.5)	18 (56.2)	1.000	
*CEP72* rs924607 C > T	C	17 (77.3)	47 (73.4)	0.785	0.07
	T	5 (22.7)	17 (26.6)		
	CC	7 (63.6)	15 (46.9)	0.488	
	CT	3 (27.3)	17 (53.1)	0.175	
	TT	1 (9.1)	0 (0.0)	0.259	
*CEP72* rs12522955 C > A	C	18 (81.8)	51 (79.7)	1.000	0.58
	A	4 (18.2)	13 (20.3)		
	CC	7 (63.6)	21 (65.7)	1.000	
	CA	4 (36.4)	9 (28.1)	0.709	
	AA	0 (0.0)	2 (6.2)	1.000	

Values are presented as n (%). ^1^ Fisher’s exact test. ^2^ HWE: Hardy–Weinberg equilibrium assessed in the control group by exact test. The bold values represent statistically significant *p*-values (*p* < 0.05).

**Table 3:** Inheritance models for *ABCC2* rs11190298 and rs3740066 (n = 43).

SNV	Model	Cases n = 11	Controls n = 32	OR	95%CI	*p*-Value	AIC
	Codominant						
*ABCC2* rs11190298	A/A	6 (54.5)	5 (15.6)	1.00			
	A/G	5 (45.5)	20 (62.5)	0.21	0.04–0.97	**0.013**	46.2
	G/G	0 (0.0)	7 (21.9)	NA	0.00-NA		
	Dominant						
	A/A	6 (54.5)	5 (15.6)	1.00			
	A/G-G/G	5 (45.5)	27 (84.4)	0.15	0.03–0.71	**0.014**	46.9
	Recessive						
	A/A-A/G	11 (100)	25 (78.1)	1.00			
	G/G	0 (0.0)	7 (21.9)	NA	0.00-NA	**0.032**	48.3
	Log-additive			0.17	0.04–0.66	**0.004**	44.8
	Codominant						
*ABCC2* rs3740066	C/C	6 (54.5)	8 (25.0)	1.00			
	C/T	5 (45.5)	19 (59.4)	0.35	0.08–1.49	0.074	49.7
	T/T	0 (0.0)	5 (15.6)	NA	0.00-NA		
	Dominant						
	C/C	6 (54.5)	8 (25.0)	1.00			
	C/T-T/T	5 (45.5)	24 (75.0)	0.28	0.07–1.16	0.077	49.8
	Recessive						
	C/C-C/T	11 (100)	27 (84.4)	1.00			
	T/T	0 (0.0)	5 (15.6)	NA	0.00-NA	0.075	49.7
	Log-additive			0.28	0.08–0.99	**0.034**	48.4

OR: Odds Ratio; 95% CI: 95% Confidence Interval; AIC: Akaike Information Criterion. The bold values represent statistically significant *p*-values (*p* < 0.05).

**Table 4:** Haplotype association of *ABCC2* with VIPN (n = 43).

ABCC2 rs11190298	ABCC2 rs3740066	Frequency	OR (95% CI)	*p*-Value
A	C	0.4614	1.00	---
G	T	0.3103	0.06 (0.01–0.55)	0.018
G	C	0.1432	0.03 (0.00–0.53)	0.022
A	T	0.0851	0.04 (0.00–0.88)	0.048
Global haplotype association *p*-value: 0.003				

Haplotypes were estimated by the EM algorithm in SNPStats; ORs are relative to the most frequent haplotype (A–C). Wide confidence intervals reflect sparse data.

## Data Availability

De-identified individual-level genotype data and clinical variables supporting the reported results are available from the corresponding authors upon reasonable request, subject to ethics committee approval and protection of participant confidentiality.

## Supplementary Materials

The following supporting information can be downloaded at https://www.mdpi.com/article/10.3390/ijms27219998/s1.

## Abbreviations

The following abbreviations are used in this manuscript:

ALL	Acute lymphoblastic leukemia
VIPN	Vincristine-induced peripheral neuropathy
SNV	Single-nucleotide variant
CTCAE	Common Terminology Criteria for Adverse Events

## References

[1] Brown P., Inaba H., Annesley C., Beck J., Colace S., Dallas M., DeSantes K., Kelly K., Kitko C., Lacayo N. (2020). Pediatric Acute Lymphoblastic Leukemia, Version 2.2020, NCCN Clinical Practice Guidelines in Oncology. J. Natl. Compr. Canc. Netw..

[2] Rivera-Luna R., Zapata-Tarres M., Shalkow-Klincovstein J., Velasco-Hidalgo L., Olaya-Vargas A., Finkelstein-Mizrahi N., Cárdenas-Cardós R., Aguilar-Ortiz M.R. (2017). The Burden of Childhood Cancer in Mexico: Implications for Low- and Middle-Income Countries. Pediatr. Blood Cancer.

[3] Škubník J., Pavlíčková V.S., Ruml T., Rimpelová S. (2021). Vincristine in Combination Therapy of Cancer: Emerging Trends in Clinics. Biology.

[4] Verma P., Devaraj J., Skiles J.L., Sajdyk T., Ho R.H., Hutchinson R., Wells E., Li L., Renbarger J., Cooper B. (2020). A Metabolomics Approach for Early Prediction of Vincristine-Induced Peripheral Neuropathy. Sci. Rep..

[5] Dennison J.B., Jones D.R., Renbarger J.L., Hall S.D. (2007). Effect of CYP3A5 Expression on Vincristine Metabolism with Human Liver Microsomes. J. Pharmacol. Exp. Ther..

[6] Egbelakin A., Ferguson M.J., MacGill E.A., Lehmann A.S., Topletz A.R., Quinney S.K., Li L., McCammack K.C., Hall S.D., Renbarger J.L. (2011). Increased Risk of Vincristine Neurotoxicity Associated with Low CYP3A5 Expression Genotype in Children with Acute Lymphoblastic Leukemia. Pediatr. Blood Cancer.

[7] Skiles J.L., Chiang C., Li C.H., Martin S., Smith E.L., Olbara G., Jones D.R., Vik T.A., Mostert S., Abbink F. (2018). CYP3A5 Genotype and Its Impact on Vincristine Pharmacokinetics and Development of Neuropathy in Kenyan Children with Cancer. Pediatr. Blood Cancer.

[8] Dennison J.B., Kulanthaivel P., Barbuch R.J., Renbarger J.L., Ehlhardt W.J., Hall S.D. (2006). Selective Metabolism of Vincristine In Vitro by CYP3A5. Drug Metab. Dispos..

[9] Aplenc R., Glatfelter W., Han P., Rappaport E., La M., Cnaan A., Blackwood M.A., Lange B., Rebbeck T. (2003). CYP3A Genotypes and Treatment Response in Paediatric Acute Lymphoblastic Leukaemia. Br. J. Haematol..

[10] Lopez-Lopez E., Gutierrez-Camino A., Astigarraga I., Navajas A., Echebarria-Barona A., Garcia-Miguel P., Garcia De Andoin N., Lobo C., Guerra-Merino I., Martin-Guerrero I. (2016). Vincristine Pharmacokinetics Pathway and Neurotoxicity During Early Phases of Treatment in Pediatric Acute Lymphoblastic Leukemia. Pharmacogenomics.

[11] Yuan Y., Hu W., Chen C., Yao R., Zhang S., Zhu X., Xu B., Huang Z., Zhang S., Wang X. (2025). Pharmacokinetic, Pharmacodynamic and Pharmacogenetic Studies Related to Vincristine-Induced Peripheral Neuropathy in Chinese Pediatric ALL Patients. Clin. Pharmacol. Ther..

[12] Zgheib N.K., Ghanem K.M., Tamim H., Aridi C., Shahine R., Tarek N., Saab R., Abboud M.R., El-Solh H., Muwakkit S.A. (2018). Genetic Polymorphisms in Candidate Genes Are Not Associated with Increased Vincristine-Related Peripheral Neuropathy in Arab Children Treated for Acute Childhood Leukemia: A Single Institution Study. Pharmacogenet. Genom..

[13] Diouf B., Crews K.R., Lew G., Pei D., Cheng C., Bao J., Zheng J.J., Yang W., Fan Y., Wheeler H.E. (2015). Association of an Inherited Genetic Variant with Vincristine-Related Peripheral Neuropathy in Children with Acute Lymphoblastic Leukemia. JAMA.

[14] Klumpers M.J., Brand A.C.A.M., Hakobjan M., Gattuso G., Schiavello E., Terenziani M., Massimino M., Gidding C.E.M., Guchelaar H., Te Loo D.M.W.M. (2022). Contribution of Common and Rare Genetic Variants in CEP72 on Vincristine-Induced Peripheral Neuropathy in Brain Tumour Patients. Br. J. Clin. Pharmacol..

[15] Gonzalez-Covarrubias V., Morales-Franco M., Cruz-Correa O.F., Martínez-Hernández A., García-Ortíz H., Barajas-Olmos F., Genis-Mendoza A.D., Martínez-Magaña J.J., Nicolini H., Orozco L. (2019). Variation in Actionable Pharmacogenetic Markers in Natives and Mestizos from Mexico. Front. Pharmacol..

[16] Vargas-Alarcón G., Ramírez-Bello J., De La Peña A., Calderón-Cruz B., Peña-Duque M.A., Martínez-Ríos M.A., Ramírez-Fuentes S., Pérez-Méndez O., Fragoso J.M. (2014). Distribution of ABCB1, CYP3A5, CYP2C19, and P2RY12 Gene Polymorphisms in a Mexican Mestizos Population. Mol. Biol. Rep..

[17] Rokkanen R., Aarnivala H., Pokka T., Niinimäki R. (2024). Chemotherapy-Related Toxicities Follow a Typical Pattern in Children Treated for Acute Lymphoblastic Leukaemia. Acta Paediatr..

[18] Alvarado-Sánchez R. (2015). Frecuencia de Neuropatía Periférica Secundaria a Vincristina en Pacientes Pediátricos con Leucemia Linfoblástica Aguda. Especialidad en Pediatría Thesis.

[19] Vivas-Rosales I.J., Garcia-Saavedra L., Martin-Trejo J.A., Mejia-Arangure J.M., Victorio-Garcia N.P., Herrera-Landero A., Núñez-Enríquez J.C. (2017). Chemotherapy-Induced Peripheral Neuropathy in a Subpopulation of Mexican Pediatric Patients with Acute Lymphoblastic Leukemia. Iran. J. Pediatr..

[20] Gutierrez-Camino A., Martin-Guerrero I., Lopez-Lopez E., Echebarria-Barona A., Zabalza I., Ruiz I., Guerra-Merino I., Garcia-Orad A. (2016). Lack of Association of the CEP72 Rs924607 TT Genotype with Vincristine-Related Peripheral Neuropathy During the Early Phase of Pediatric Acute Lymphoblastic Leukemia Treatment in a Spanish Population. Pharmacogenet. Genom..

[21] Nama N., Barker M.K., Kwan C., Sabarre C., Solimano V., Rankin A., Raabe J., Ross C.J., Carleton B., Zwicker J.G. (2020). Vincristine-Induced Peripheral Neurotoxicity: A Prospective Cohort. Pediatr. Hematol. Oncol..

[22] Tunjungsari D.A., Gunawan P.I., Ugrasena I.D.G. (2021). Risk Factors of Vincristine-Induced Peripheral Neuropathy in Acute Lymphoblastic Leukaemia Children. J. Med. Invest..

[23] Martin-Guerrero I., Gutierrez-Camino A., Echebarria-Barona A., Astigarraga I., Garcia De Andoin N., Navajas A., Garcia-Orad A. (2019). Variants in Vincristine Pharmacodynamic Genes Involved in Neurotoxicity at Induction Phase in the Therapy of Pediatric Acute Lymphoblastic Leukemia. Pharmacogenomics J..

[24] Yang Q.-Y., Hu Y.-H., Guo H.-L., Xia Y., Zhang Y., Fang W.-R., Li Y.-M., Xu J., Chen F., Wang Y.-R. (2021). Vincristine-Induced Peripheral Neuropathy in Childhood Acute Lymphoblastic Leukemia: Genetic Variation as a Potential Risk Factor. Front. Pharmacol..

[25] Guilhaumou R., Simon N., Quaranta S., Verschuur A., Lacarelle B., Andre N., Solas C. (2011). Population Pharmacokinetics and Pharmacogenetics of Vincristine in Paediatric Patients Treated for Solid Tumour Diseases. Cancer Chemother. Pharmacol..

[26] Hartman A., Van Schaik R.H.N., Van Der Heiden I.P., Broekhuis M.J.C., Meier M., Den Boer M.L., Pieters R. (2010). Polymorphisms in Genes Involved in Vincristine Pharmacokinetics or Pharmacodynamics Are Not Related to Impaired Motor Performance in Children with Leukemia. Leuk. Res..

[27] Kavcic M., Koritnik B., Krzan M., Velikonja O., Prelog T., Stefanovic M., Debeljak M., Jazbec J. (2017). Electrophysiological Studies to Detect Peripheral Neuropathy in Children Treated with Vincristine. J. Pediatr. Hematol. Oncol..

[28] Diouf B., Crews K.R., Evans W.E. (2016). Vincristine Pharmacogenomics: ‘Winner’s Curse’ or a Different Phenotype?. Pharmacogenet. Genom..

[29] Martínez-Cortés G., Salazar-Flores J., Fernández-Rodríguez L.G., Rubi-Castellanos R., Rodríguez-Loya C., Velarde-Félix J.S., Muñoz-Valle J.F., Parra-Rojas I., Rangel-Villalobos H. (2012). Admixture and Population Structure in Mexican-Mestizos Based on Paternal Lineages. J. Hum. Genet..

[30] Van De Velde M.E., Uittenboogaard A., Yang W., Bonten E., Cheng C., Pei D., Van Den Berg M.H., Van Der Sluis I.M., Van Den Bos C., Abbink F.C.H. (2022). Genetic Polymorphisms Associated with Vincristine Pharmacokinetics and Vincristine-Induced Peripheral Neuropathy in Pediatric Oncology Patients. Cancers.

[31] Iranpur M., Esmailizadeh A. Rapid Extraction of High Quality DNA from Whole Blood Stored at 4 °C for Long Period. Protocol Online.

[32] Solé X., Guinó E., Valls J., Iniesta R., Moreno V. (2006). SNPStats: A Web Tool for the Analysis of Association Studies. Bioinformatics.

