# Supplementary material for: ABCC2 Genetic Variation and Vincristine-Induced Peripheral Neuropathy Risk in Mexican Pediatric Patients with Acute Lymphoblastic Leukemia
Source: Int J Mol Sci. 2026 Nov 8;27(21):9998. doi: 10.3390/ijms27219998 (PMC13763606; doi:10.3390/ijms27219998)
Supplement: Supplementary file 1 [file ijms-27-09998-s001.zip › ijms-4663951-supplementary.pdf]

Supplementary Materials

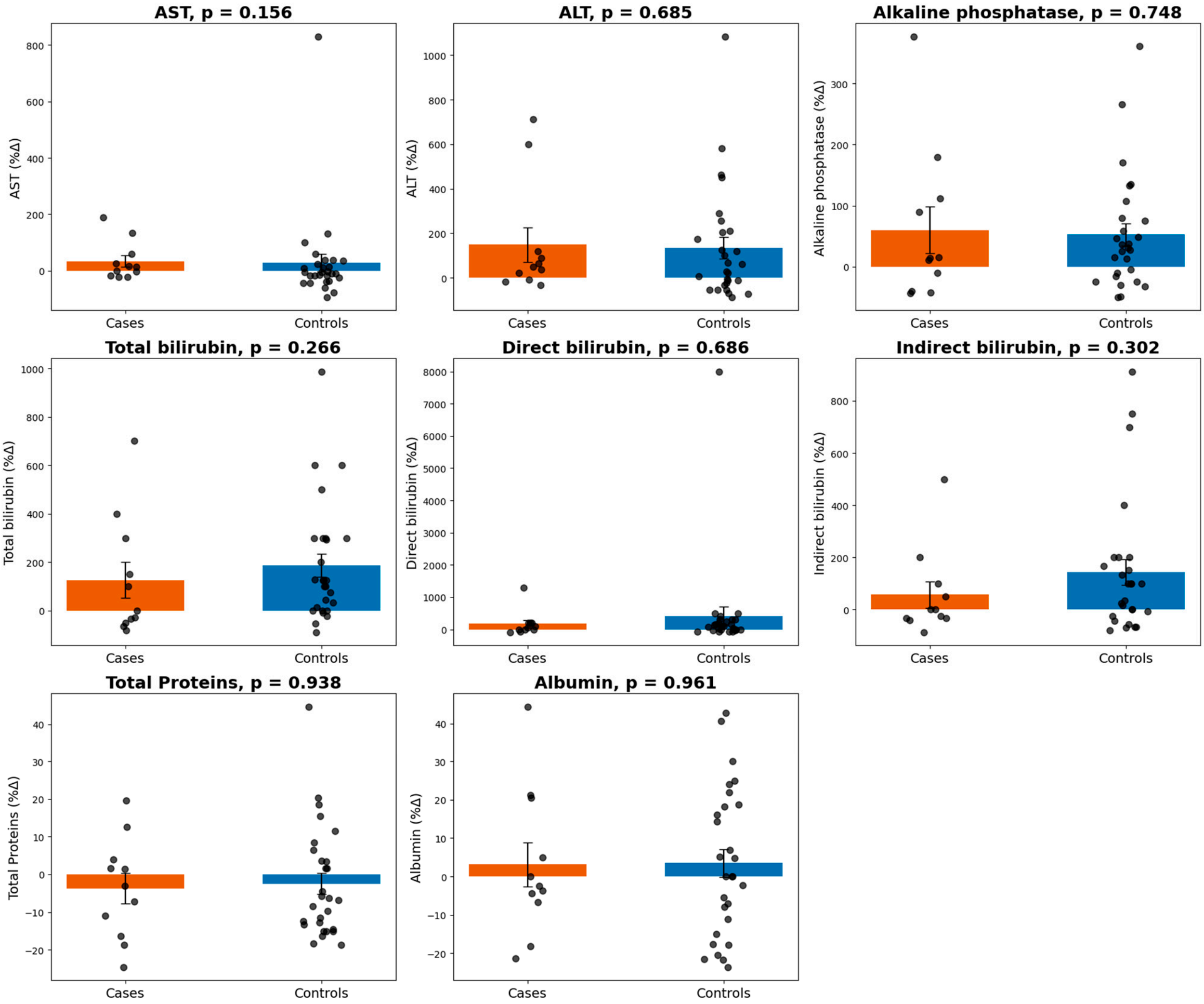

Figure S1. Relative change (Δ%) in aspartate aminotransferase (AST), alanine aminotransferase (ALT), alkaline phosphatase (ALP), total, direct and indirect bilirubin, total proteins, and albumin between cases and controls (Mann–Whitney U test, n = 43).

File S1: Vincristine-Induced Peripheral Neuropathy (VIPN) Evaluation Questionnaire

General Patient Information

1. Patient Record: ____________

2. Age at Time of Vincristine Treatment: _______ years

3. Sex: ☐ Male ☐ Female

4. Primary Oncologic Diagnosis: ________________________

5. Date Vincristine Treatment Started: ______________

6. Total Cumulative Vincristine Dose (mg/m^2): ________________

Neuropathic Symptoms Reported in Medical Record

Abnormal Sensation in Extremities

7. Was Tingling or Numbness Reported in Upper and/or Lower Extremities?

☐ No

☐ Yes, mild (occasional discomfort)

☐ Yes, moderate (frequent but does not interfere with daily activities)

☐ Yes, severe (interferes with daily activities)

Motor disturbances

8. Was weakness in the upper and/or lower extremities reported?

☐ No

☐ Yes, mild (can perform normal movements, but with slight difficulty)

☐ Yes, moderate (affects some daily activities, such as buttoning clothes or climbing stairs)

☐ Yes, severe (requires assistance for basic activities)

9. Was loss of deep tendon reflexes reported on the neurological examination?

☐ Not assessed

☐ No

☐ Yes, areflexia (absence of reflexes)

☐ Yes, hyporeflexia (decreased reflexes)

☐ Yes, hyperreflexia (increased reflexes)

Neuropathic pain

10. Was neuropathic pain reported in the extremities?

☐ No

☐ Yes, mild (occasional, does not require treatment)

☐ Yes, moderate (requires occasional analgesic treatment)

☐ Yes, severe (requires ongoing analgesic treatment)

Other symptoms associated with vincristine-induced neuropathy

11. Was jaw pain reported?

☐ No

☐ Yes, mild (occasional, does not interfere with eating)

☐ Yes, moderate (affects eating, but tolerable)

☐ Yes, severe (difficulty chewing or opening the mouth)

12. Was hoarseness or voice change reported?

☐ No

☐ Yes, mild (mild voice alteration)

☐ Yes, moderate (difficulty speaking for prolonged periods)

☐ Yes, severe (inability to speak clearly)

13. Was severe constipation reported in association with treatment?

☐ No

☐ Yes, mild (no medical intervention required)

☐ Yes, moderate (required laxatives or a special diet)

☐ Yes, severe (required hospitalization or specialized management)

Observations:

____

____

____

____

____

____

Table S1. Inheritance models for SNVs in *ABCC1, ABCB1, CYP3A5* and *CEP72.*

SNV	Model	Cases n = 11	Controls n= 32	OR	95% CI	p-value	AIC
	**Codominant**						
	C/C	6 (54.5)	15 (46.9)	1.00			
	C/T	4 (36.4)	14 (43.8)	0.71	0.17-3.08	0.90	54.7
	T/T	1 (9.1)	3 (9.4)	0.83	0.07-9.69		
	**Dominant**						
ABCC1 **rs3743527**	C/C	6 (54.5)	15 (46.9)	1.00			
	C/T –T/T	5 (45.5)	17 (53.1)	0.74	0.19-2.91	0.66	52.7
	**Recessive**						
	C/C-T/T	10 (90.9)	29 (90.6)	1.00			
	T/T	1 (9.1)	3 (9.4)	0.97	0.09-10.39	0.98	52.9
	**Log-additive**	---	---	0.83	0.28-2.42	0.73	52.8
	**Codominant**						
	A/A	2 (18.2)	9 (28.1)	1.00			
	A/C	6 (54.5)	18 (56.2)	1.50	0.25-8.98	0.64	54.0
ABCB1 **rs2032582**	C/C	3 (27.3)	5 (15.6)	2.70	0.33-21.98		
	**Dominant**						
	A/A	2 (18.2)	9 (28.1)	1.00			
	A/C –C/C	9 (81.8)	23 (71.9)	1.76	0.32-9.79	0.5	52.5

	**Recessive**						
	A/A-A/C	8 (72.7)	27 (84.4)	1.00			
	C/C	3 (27.3)	5 (15.6)	2.02	0.40-10.38	0.41	52.2
	**Log-additive**			1.65	0.57-4.76	0.35	52.0
ABCB1 **rs1045642**	**Codominant**						
	G/G	3 (27.3)	6 (18.8)	1.00			
	A/G	6 (54.5)	22 (68.8)	0.55	0.10-2.85	0.70	54.2
	A/A	2 (18.2)	4 (12.5)	1.00	0.11-8.95		
	**Dominant**						
	G/G	3 (27.3)	6 (18.8)	1.00			
	A/G –A/A	8 (72.7)	26 (81.2)	0.62	0.12-3.04	0.56	52.6
	**Recessive**						
	G/G-A/G	9 (81.8)	28 (87.5)	1.00			
	A/A	2 (18.2)	4 (12.5)	1.56	0.24-9.95	0.65	52.7
	**Log-additive**			0.92	0.29-2.96	0.89	52.9
CYP3A5 rs776746	**Codominant**						
	G/G	6 (54.5)	18 (56.2)	1.00			
	A/G	4 (36.4)	12 (37.5)	1.00	0.23-4.31	0.95	54.8
	A/A	1 (9.1)	2 (6.2)	1.50	0.11-19.64		
	**Dominant**						
	G/G	6 (54.5)	18 (56.2)	1.00			

	A/G-A/A	5 (45.5)	14 (43.8)	1.07	0.27-4.25	0.92	52.9
	**Recessive**						
	G/G-A/G	10 (90.9)	30 (93.8)	1.00			
	A/A	1 (9.1)	2 (6.2)	1.50	0.12-18.36	0.76	52.8
	**Log-additive**			1.12	0.38-3.32	0.84	52.9
***CEP72* rs924607**	**Codominant**						
	C/C	7 (63.6)	15 (46.9)	1.00			
	C/T	3 (27.3)	17 (53.1)	0.38	0.08-1.73	0.11	50.4
	T/T	1 (9.1)	0 (0.0)	0.00	0.00-NA		
	**Dominant**						
	C/C	7 (63.6)	15 (46.9)	1.00			
	C/T- T/T	4 (36.4)	17 (53.1)	0.50	0.12-2.07	0.33	52.0
	**Recessive**						
	C/C-C/T	10 (90.9)	32 (100)	1.00			
	T/T	1 (9.1)	0 (0.0)	0.00	0.00-NA	0.09	50.1
	**Log-additive**			0.77	0.21-2.77	0.69	52.7
***CEP72* rs12522955**	**Codominant**						
	C/C	7 (63.6)	21 (65.6)	1.00			
	C/A	4 (36.4)	9 (28.1)	1.33	0.31-5.72	0.51	53.5
	A/A	0 (0.0)	2 (6.2)	NA	0.00-NA		
	**Dominant**						

C/C	7 (63.6)	21 (65.6)	1.00			
C/A-A/A	4 (36.4)	11 (34.4)	1.09	0.26-4.55	0.91	52.9
**Recessive**						
C/C-C/A	11 (100)	30 (93.8)	1.00			
A/A	0 (0.0)	2 (6.2)	NA	0.00-NA	0.27	51.7
**Log-additive**			0.88	0.26-2.96	0.83	52.9

OR: Odds Ratio; CI: 95% Confidence Interval; AIC: Akaike Information Criterion

Table S2. Haplotype association of *ABCB1* with VIPN (n = 43).

ABCB1 rs2032582	*ABCB1* rs1045642	Frequency	OR (95% CI)	p-value
A	A	0.4638	1.00	---
C	G	0.4522	1.30 (0.39-4.30)	0.67
A	G	0.0711	0.00	1
C	A	0.0129	0.00	1
Global haplotype association p-value: 0.25				

Table S3. Haplotype association of *CYP3A* with VIPN (n = 43).

CYP3A5 rs776746	*CYP3A4* rs2740574	Frequency	OR (95% CI)	p-value
G	T	0.7105	1.00	---
A	T	0.2197	0.87 (0.26-2.89)	0.82
A	C	0.0361	5.28 (0.20-139.93)	0.33

G	C	0.0337	0.00	1
Global haplotype association p-value: 0.5				

Table S4. Haplotype association of *CEP72* with VIPN (n = 43).

***CEP72* rs924607**	***CEP72* rs12522955**	**Frequency**	**OR (95% CI)**	**p-value**
C	C	0.5465	1.00	---
T	C	0.2558	0.72 (0.19 – 2.73)	0.64
C	A	0.1977	0.81 (0.23 – 2.85)	0.74
Global haplotype association p-value: 0.87				
